# Longitudinal FDG-PET Radiomics for Early Prediction of Treatment Response to Chemoradiation in Locally Advanced Cervical Cancer: A Pilot Study

**DOI:** 10.3390/cancers16223813

**Published:** 2024-11-13

**Authors:** Alejandro Cepero, Yidong Yang, Lori Young, Jianfeng Huang, Xuemei Ji, Fei Yang

**Affiliations:** 1 Department of Biomedical Engineering, University of Miami, Coral Gables, FL 33146, USA; 2 The First Affiliated Hospital of the University of Science and Technology of China, Hefei 230001, China; 3 Department of Radiation Oncology, University of Washington School of Medicine, Seattle, WA 98195, USA; 4 Department of Radiation Oncology, Affiliated Hospital of Jiangnan University, Wuxi 214122, China; 5 Department of Therapeutic Radiology, Yale School of Medicine, New Haven, CT 06520, USA; 6 Department of Radiation Oncology, University of Miami School of Medicine, Miami, FL 33136, USA

**Keywords:** LACC, chemoradiation, radiomics, PET, predictive modeling, personalized medicine

## Abstract

**Objectives**: This study aimed to assess the capacity of longitudinal FDG-PET radiomics for early distinguishing between locally advanced cervical cancer (LACC) patients who responded to treatment and those who did not. **Methods**: FDG-PET scans were obtained before and midway through concurrent chemoradiation for a study cohort of patients with LACC. Radiomics features related to image textures were extracted from the primary tumor volumes and stratified for relevance to treatment response status with the aid of random forest recursive feature elimination. Predictive models based on the k-nearest neighbors time series classifier were developed using the top-selected features to differentiate between responders and non-responders. The performance of the developed models was evaluated using receiver operating characteristic (ROC) curve analysis and n-fold cross-validation. **Results**: The top radiomics features extracted from scans taken midway through treatment showed significant differences between the two responder groups (*p*-values < 0.0005). In contrast, those from pretreatment scans did not exhibit significant differences. The AUC of the mean ROC curve for the predictive model based on the top features from pretreatment scans was 0.8529, while it reached 0.9420 for those derived midway through treatment scans. **Conclusions**: The study highlights the potential of longitudinal FDG-PET radiomics extracted midway through treatment for predicting response to chemoradiation in LACC patients and emphasizes that interim PET scans could be crucial in personalized medicine, ultimately enhancing therapeutic outcomes for LACC.

## Simple Summary

Locally advanced cervical cancer (LACC) poses a significant challenge in oncology due to its heterogeneous nature and inconsistent response to standard chemoradiation therapy. The ability to predict response to treatment early in LACC has profound implications for enhancing patient care through tailored and adaptive therapies. Sequential FDG-PET imaging is crucial in managing LACC, offering a non-invasive method to assess tumor progression and response to chemoradiation over the course of treatment. The current study had recourse to longitudinal FDG-PET radiomics for early prediction of treatment response to chemoradiation in LACC. The implications of this study aid in demonstrating the potentially pivotal role of longitudinal radiomics in personalized medicine and improving therapeutic outcomes for LACC.

## 1. Introduction

Cervical cancer is estimated to be the fourth most common cancer among women worldwide, with approximately 570,000 new cases and 311,000 deaths in 2018 [1]. Due mainly to the lack of regular screening, restricted healthcare access, and disparities in healthcare, among many other socioeconomic factors, a substantial portion of cases were identified at an advanced stage, which limits treatment options and decreases the likelihood of cure [1]. At advanced stages, the disease typically infiltrates nearby tissues and spreads to lymph nodes or other organs. Treatment often requires a multimodal approach, combining surgery, radiation therapy, and chemotherapy, among different regimes. However, treatment failure is expected. For patients with these advanced stages of cervical cancer, up to a third have a recurrence within 18 months of therapy, and the 5-year survival rate is 65% for International Federation of Gynecology and Obstetrics (FIGO) stage II, 40% for stage III, and only 15% for stage IV [2].

^18^Fluorodeoxyglucose (^18^F-FDG) Positron Emission Tomography (PET), capturing the metabolic activity within the tumor and allowing for intra-tumoral heterogeneity characterization, plays a vital role in many aspects of the management of LACC [3,4]. There has been evidence to suggest that FDG-PET can be influential in the diagnosis and staging of LACC, given that it allows to accurately determine the extent of disease by identifying the presence of metastases and assessing lymph node involvement in a non-invasive manner [5]. As for treatment planning in LACC, when combined with other genetic and clinical information, FDG-PET can contribute to the development of individualized treatment regimens tailored to the patient’s specific tumor characteristics [6,7]. FDG-PET is also beneficial for monitoring treatment responses in LACC, potentially enabling timely modifications to treatment strategies as needed to enhance therapeutic advantages [5,6,8]. In the post-treatment setting, FDG-PET is also routinely resorted to for surveillance to detect recurrence and for assessment of residual disease, thus guiding subsequent treatment decisions [6].

Radiomics is an emerging field focused on extracting quantitative radiographic features to transform digital medical images into high-dimensional data, which are then analyzed to support clinical decision-making [9]. Radiomics features derived from FDG-PET imaging, in contrast to anatomical modalities like CT and MRI, can capture temporospatial metabolic and functional variations within tumors. This provides a more detailed understanding of the heterogeneity in cancerous tissues, often before structural changes are visible [4]. As such, these features are considered surrogate markers for the underlying phenotypic and genetic heterogeneity of tumors, which are believed to be linked to their sensitivity to therapies [10]. FDG-PET radiomics has already demonstrated potential in improving the precision of diagnosis, prognosis, and treatment for various cancers, including those of the head and neck, lung, endometrium, stomach, rectum, and many others [11,12,13,14,15].

In the context of LACC, PET radiomics has made significant inroads in its potential to predict response to chemoradiation therapy. Studies have shown that specific PET radiomic features can be correlated with responses to treatment. For example, Burchardt et al. employed baseline FDG-PET radiomics to predict treatment response in LACC. The study demonstrated that metabolic activity features extracted from PET images could predict recurrence and response, allowing for early treatment modification, if necessary [16]. Lucia et al. used radiomics features from pretreatment FDG-PET scans to predict overall survival in LACC patients. They found that features related to tumor heterogeneity and metabolic activity were strong predictors of survival, outperforming traditional clinical and pathological parameters [17]. Additionally, Ferreira explored the use of PET radiomics to predict disease-free survival in LACC patients. Their findings highlighted that radiomic features provide added values to the standard clinical variables in terms of predicting outcomes in LACC [18]. However, most of these studies have focused on radiomics features derived from pretreatment PET images, with, to our knowledge, very limited research exploring radiomics features from PET data obtained during the course of treatment. While baseline imaging and its associated radiomic features provide a crucial reference point for assessing the initial extent of the disease, they lack insights into how the tumor would respond to treatment.

In the current study, we hypothesized that radiomics parameters derived from interim PET imaging taken midway through the treatment offer enhanced predictive value for treatment responses to chemotherapy in LACC compared to those extracted from baseline imaging. To validate this hypothesis, the current study assessed and compared the predictive power of radiomics features derived from interim scans acquired midway through the treatment course with those from the baseline scans in a cohort of LACC patients undergoing longitudinal PET imaging during chemoradiation.

## 2. Materials and Methods

### 2.1. PET Imaging and Patient Population

The current study made use of patient data obtained from the Cervical Cancer Tumor Heterogeneity (CCTH) collection, with the imaging and clinical data publicly accessible from The Cancer Imaging Archive (TCIA) [19,20]. The local institutional review board (IRB) determined that ethical approval was unnecessary for this study, given that only publicly available aggregate patient data were utilized. This collection comprised 23 LACC patients with FIGO stage IIB to IVA. Each of the included patients had received a combined course of external beam radiation therapy, concurrent weekly cisplatin-based chemotherapy, and subsequent ^192^Ir high dose rate (HDR) intracavitary brachytherapy for curative intent. External beam RT comprised whole pelvic irradiation followed by cone-down boosts to the gross disease with prescription doses ranging from 45.0–50.4 Gy in 1.8–2 Gy per fraction. Chemotherapy consisted of weekly cisplatin administration at a dose of 40 mg/m^2^ for six cycles. HDR brachytherapy was provided towards the end of or following the completion of external beam radiation with a biologically equivalent dose in 2 Gy (α/β = 10 Gy) ranging from 30.0–39.6 Gy [21].

PET/CT images were acquired, following a standardized multi-institutional protocol, from two scanners (GE Discovery STE PET/CT system and Siemens Biograph 64 TP PET/CT system) at three distinct time points: before treatment initiation, early during the treatment course (2–2.5 weeks post-treatment initiation), and midway during treatment course (4–5 weeks post-treatment initiation). Each PET/CT exam started with a helical low-dose CT scan for attenuation correction and identification of anatomical landmarks, followed by a whole-body static PET acquisition covering 6–7 axial fields of view with an imaging time of 5 min on average per bed position [19]. Patients fasted for at least 4 h before receiving weight-dependent FDG injection, and the uptake period before scanning averaged around 1 h. All PET images were reconstructed by the ordered subset expectation maximization (OSEM) algorithm followed by a post-reconstruction smoothing Gaussian filter [21].

### 2.2. Radiomics Analysis and Feature Extraction

It is well established that radiation-induced inflammation peaks around 2 to 3 weeks into a radiotherapy course [22,23]. This inflammation can lead to increased FDG uptake on PET scans, potentially causing false positives and complicating the evaluation of treatment responses [24]. To avoid this confounding factor, PET scans taken early in the treatment course (2–2.5 weeks after treatment initiation) were excluded from the analysis. As such, this study assessed three sets of radiomics data for their predictive value in response to chemoradiation for LACC. The first set comprised radiomics data extracted from pretreatment PET scans, the second comprised the interim scans acquired midway through the treatment course (4–5 weeks post-treatment initiation), and a third set combined both datasets.

The CCTH data collection included tumor volume contours, defined using the gold standard of T2-weighted MRI in conjunction with PET/CT co-registration [19]. Prior to conducting the radiomics analysis, the pre-existing contours of the primary tumor volume on the PET scans were thoroughly reviewed by a radiation oncology physician with extensive clinical experience in delineating cervical lesions for radiotherapy treatment planning, in accordance with the latest guidelines for target delineation in cervical cancer [25]. It is important to note that only primary tumor volumes were included in the radiomics analysis, as lymph node involvement varied among the patients in the study cohort. After completing the review, the PET images and their corresponding primary tumor volume contours were resampled to an isotropic voxel size of 1 mm. The gray-level values were discretized with a fixed bin width of 0.1 standard uptake value (SUV), as recommended by previous studies [26,27].

A total of 59 radiomics features were considered, including 24 derived from the gray-level co-occurrence matrix (GLCM) with a voxel displacement of 1, 16 from the gray-level run length matrix (GLRLM) without distance weighting, 5 from the neighborhood gray-tone difference matrix (NGTDM) with a neighborhood size of 3 × 3 × 3, and 14 from the gray-level dependence matrix (GLDM) with a dependence threshold of 0 and a neighbor distance of 1 [28,29,30,31,32,33]. Features based on GLCM are designed to capture the spatial relationships between voxel pairs by analyzing how often pairs of voxels with specific intensity values occur in a given spatial configuration. These features focus on local texture patterns and provide insights into the spatial distribution of gray levels, helping to characterize the heterogeneity of the image content at a finer, localized level. Radiomics features extracted from GLRLM describe the texture of an image by quantifying consecutive voxels that share the same gray-level intensity along a specific direction. These features capture regional texture patterns, such as the length and frequency of homogeneous runs of voxels, providing information on the spatial distribution of contiguous regions with similar intensity values. NGTDM features are based on how human vision perceives differences in gray levels. These features compare the gray level of each voxel with the average gray level of its neighboring voxels within a predefined distance. NGTDM captures spatial details such as coarseness, busyness, and complexity, which reflect how rapidly gray-level intensities change across an image. GLDM-based features measure the spatial relationships between voxels by quantifying how many neighboring voxels within a region of interest share the same gray-level intensity as a central voxel. These features provide insights into texture heterogeneity by assessing dependencies between neighboring voxels. Each of these feature sets provides complementary information about tumor characteristics, particularly in terms of metabolic heterogeneity, which are crucial for understanding and differentiating tumor behaviors. Following extraction, the raw data for each radiomics feature underwent normalization using standardized scores (z-scores). This process was employed to minimize the potential impact of varying scales among the features [26]. For a complete list of the radiomics features utilized, please refer to Table 1.

### 2.3. Feature Selection and Model Construction

Feature selection aims to form a compact subset of features from the extracted radiomics pool while maintaining the overall discriminatory power of the original set of features. To accomplish this, the random forest recursive feature elimination (RF-RFE) algorithm [34,35] was employed, systematically removing the least significant features based on Gini impurity and striving to pinpoint the subset of features most relevant to the endpoint, in this case, the response statute. Owing to the relatively limited number of patients in the study, for each dataset, only the top two most relevant features identified by RF-RFE were retained for the subsequent construction of predictive models.

**Figure 1:**
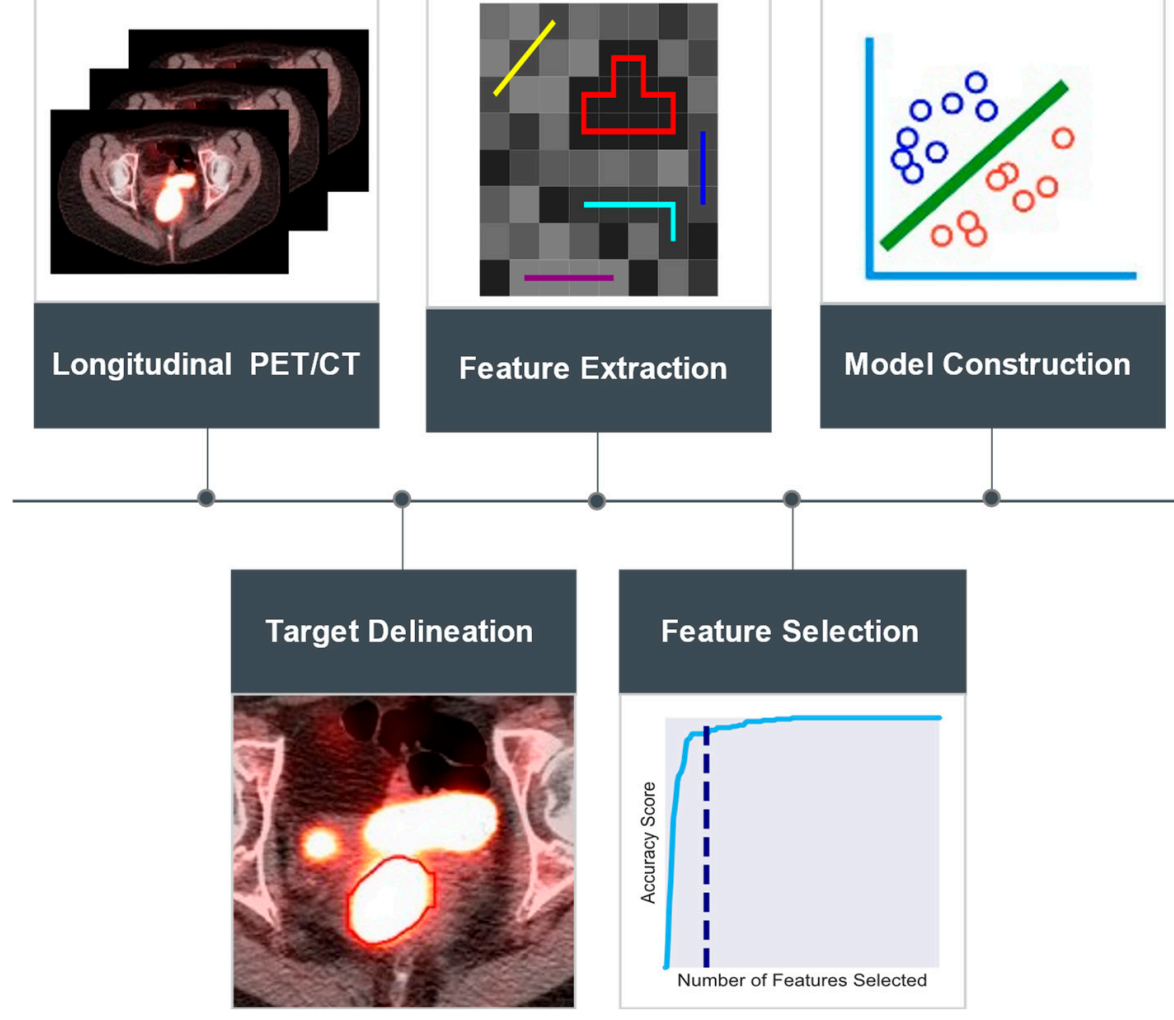
Workflow of the key steps in radiomics analysis using sequential FDG-PET scans for early prediction of chemoradiation response in locally advanced cervical cancer (LACC). Longitudinal FDG-PET scans were acquired for each patient in the study cohort before treatment initiation, early during the treatment course (2–2.5 weeks post-treatment initiation), and midway through the treatment course (4–5 weeks post-treatment initiation). For each patient, primary tumor volumes were delineated on the PET scans obtained, excluding those acquired early during treatment due to inflammation confoundment. Radiomics features were then extracted and selected using random forest recursive feature elimination (RFRFE) to identify the subset of features most relevant to the response class. Subsequently, the selected features were used to construct k-nearest neighbors (k-NN) time series classifiers.

The k-nearest neighbors (k-NN) time series classifier was utilized in this study to distinguish between responders and non-responders. This classifier is a variant of the traditional k-NN algorithm tailored for time series data. It identifies top similar time series in a training set to a given test instance and predicts the class label based on the majority class among these nearest neighbors [36]. Given the relatively small size of the study cohort, n-fold cross-validation [37] was used to build the classification models. For each set of radiomics data, the top two selected features were randomly split into four subsets. Each model was trained and validated four times, using one of the subsets for validation and the remaining three for training. This entire cross-validation process for each model was repeated 50 times with different random shuffling to minimize the impact of potential biases in data splitting. A summary of the key methodological steps is provided in Figure 1.

### 2.4. Statistical Analysis

The selected radiomics features were compared between the two responder groups using the Kruskal–Wallis test, with the null hypothesis assuming that the median feature values were equal across both cohorts [38]. The performance of the k-NN time series classifiers, designed to differentiate between patients with and without responses, was assessed through receiver operating characteristic (ROC) curve analysis [39]. Mean ROC curves for each classifier were generated by averaging the ROC curves from each cross-validation trial. The classifiers’ discriminative ability was measured using the area under the mean ROC curve (AUC). An AUC value of 1 represents a perfect classifier; values above 0.9 are considered outstanding, between 0.8 and 0.9 are excellent, and between 0.7 and 0.8 are acceptable [40]. Discriminative performance between classifiers was also compared using DeLong et al.’s method for comparing areas under ROC curves [41]. All statistical tests were two-sided, and *p*-values of 0.05 or less, adjusted for multiple comparisons using the Bonferroni–Holm method, were considered statistically significant. All analyses were conducted using JMP Pro^®^ Version 12 statistical software (SAS Institute Inc., Cary, NC, USA).

## 3. Results

**Figure 2:**
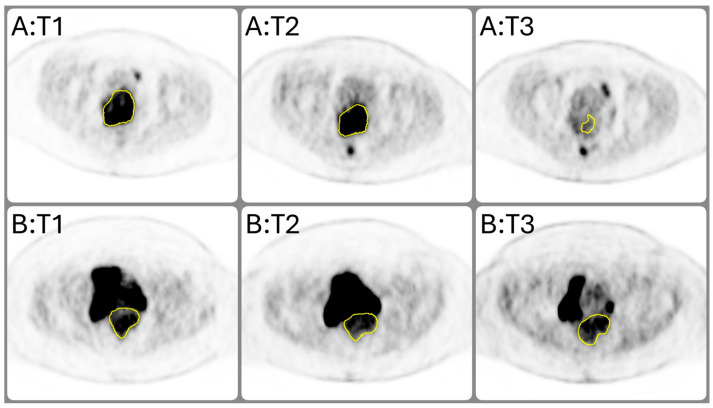
Longitudinal comparison of tumor progression and response to chemoradiation as seen in the central axial view of FDG-PET for two representative patients in the study cohort, with one who responded early (top row; **A**) and the other who did not respond (bottom row; **B**). PET scans were acquired before treatment initiation (T1), early during the treatment course (2–2.5 weeks post-treatment initiation; T2), and midway through the treatment course (4–5 weeks post-treatment initiation; T3). Primary tumor volumes at each time point are delineated in yellow.

At the time when the scans were acquired, the study cohort averaged 55 years of age, spanning from 28 to 79 years old. All presented with advanced-stage IIB–IVA epithelial carcinoma of the cervix, which included squamous cell, adeno, and undifferentiated carcinoma subtypes. Of the 23 patients included in the study, 13 responded to the treatment, while 10 did not. Figure 2 compares the longitudinal progression and response to chemoradiation at the central axial view for two lesions representative of the study cohort. The top row displays a lesion from a patient who showed an early response, while the bottom row presents a lesion from a non-responsive patient. As previously mentioned, the longitudinal PET scans were acquired before treatment initiation, early in the treatment course (2–2.5 weeks post-treatment initiation), and midway through the treatment course (4–5 weeks post-treatment initiation). At the three time points, the early responding lesion (top row) measured 104.1 cc, 74.4 cc, and 4.9 cc, whereas the non-responding lesion (bottom row) measured 123.2 cc, 90.6 cc, and 62.8 cc. Total lesion glycolysis (in SUV × cc) for the early responding lesion was calculated at 874.9, 535.7, and 15.6, compared to 490.4, 410.5, and 215.4 for the non-responding lesion across as treatment progressed.

**Figure 3:**
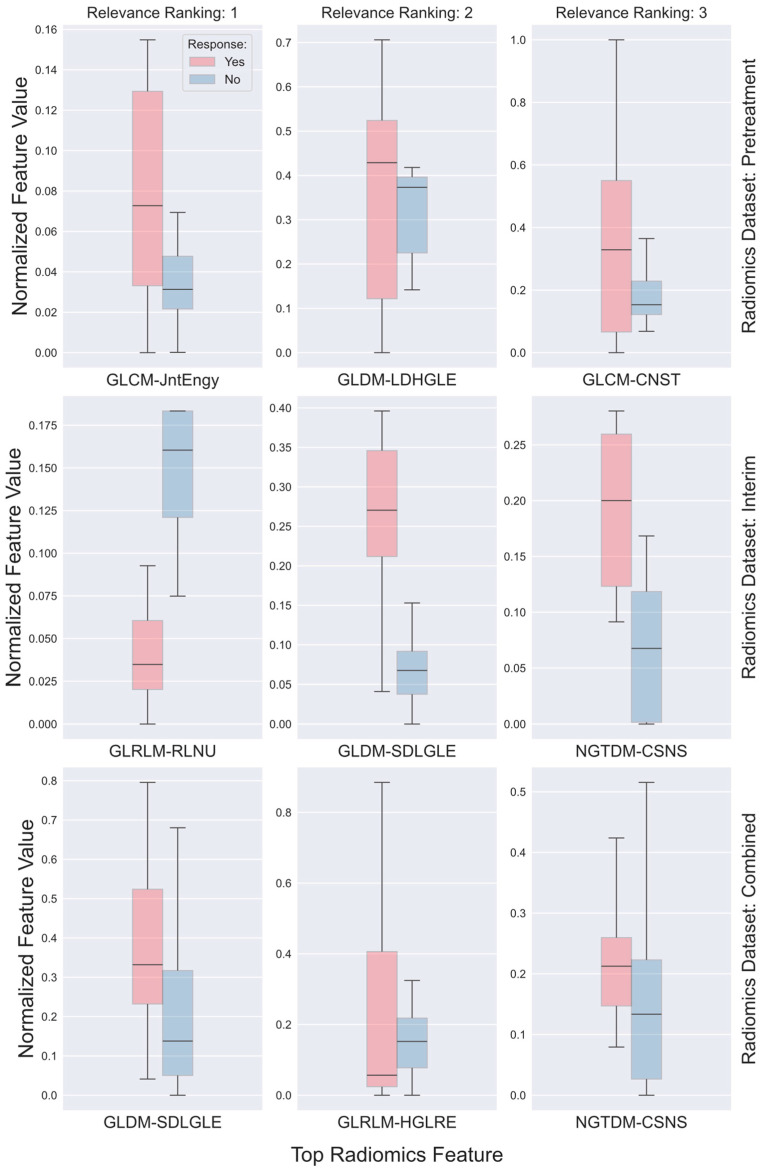
Boxplots comparing the top three features selected from radiomics datasets derived from pretreatment PET scans (**top row**), interim scans (acquired midway through treatment; **middle row**), and the combined dataset of both (**bottom row**). Radiomics feature values were normalized to a range between 0 and 1, with the vertical axis ranges adjusted to display only the data values for optimal visualization. Each box shows the median as the central mark, while the top and bottom edges represent the 25th and 75th percentiles, respectively.

Figure 3 shows the top radiomics features selected by the RF-RFE algorithm for each dataset being examined. For the first dataset, i.e., the one derived from pretreatment scans, the top three features identified by RF-RFE consist of GLCM-based joint energy (JntEngy), GLDM-based large dependence high gray-level emphasis (LDHGLE), along with GLCM-based contrast (CNST). While early responders, in general, exhibited higher values across all three radiomics features compared to those who did not respond early—with median normalized values of 0.073, 0.429, and 0.320 for GLCM-JntEngy, GLDM-LDHGLE, and GLCM-CNST, respectively, versus 0.031, 0.373, and 0.153—these features did not show significant differences between the two responder groups (*p*-values > 0.720). The top three features identified in the second dataset, which is derived from the interim scans taken midway through the treatment, are GLRLM-based run length non-uniformity (RLNU), GLDM-based small dependence low gray-level emphasis (SDLGLE), and NGTDM-based coarseness (CSNS). These features showed significant differences between the two groups (*p*-values < 0.0005), with median normalized values of 0.035, 0.270, and 0.200 for early responders, compared to 0.161, 0.068, and 0.066 for non-early responders. In the third dataset, which combines the first and second datasets, the top three features identified were GLDM-SDLGLE, GLRLM-based high gray-level run emphasis (HGLRE), and NGTDM_CSNS. The median normalized values for early responders were 0.332, 0.057, and 0.213, respectively, while for non-early responders, they were 0.138, 0.152, and 0.133. However, the differences in these features between the two responder groups did not reach statistical significance (*p*-values > 0.647).

**Figure 4:**
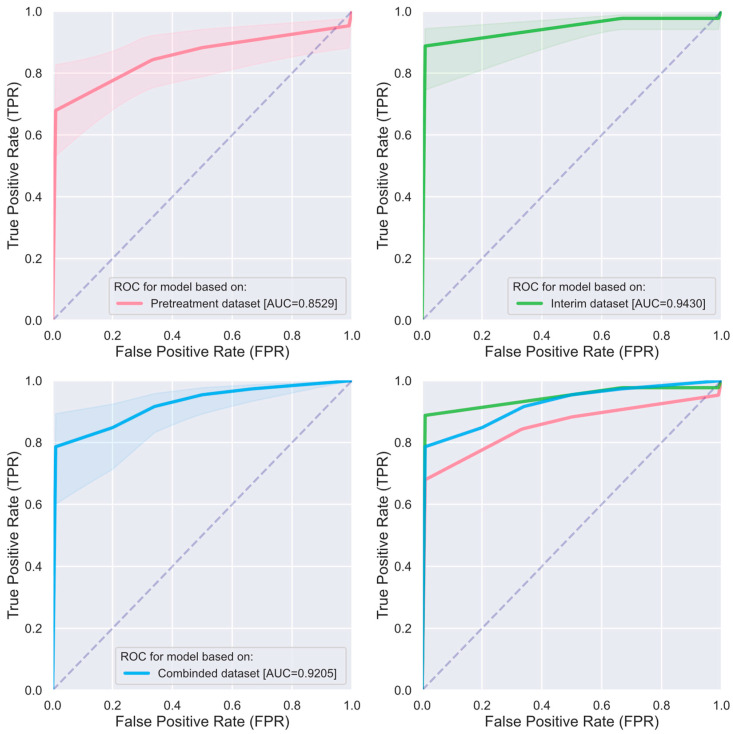
Comparison of mean receiver-operating characteristic (ROC) curves between k-NN time series classifiers built utilizing the top two radiomics features selected for radiomics dataset derived from pretreatment PET scans (in red), interim scans (in green), and the combined dataset of both (in blue) for differentiating early responders from non-responders. The diagonal dashed line from the bottom left to the top right in each diagram represents a random classifier; the closer an ROC curve is to this line, the less effective the classifier is at differentiating between the two responder groups. Shaded areas around each ROC curve indicate the 95% confidence intervals, providing an estimate of variability and reliability and indicating the range within which the model’s actual performance is likely to fall.

Figure 4 presents the mean ROC curves for the k-NN time series classifiers built using the top two features from each of the three datasets to differentiate between patients who responded to treatment and patients who did not. First, it is noted that the AUC values for all three mean ROC curves are 0.85 or higher, demonstrating that these classifiers, on average, achieved at least an excellent level of performance in distinguishing between the two patient groups. This implies that the radiomics features used in constructing these classifiers are linked to predictive power for early response to chemoradiation in patients with LACC. Additionally, DeLong test comparisons of the AUCs showed that all these classifiers significantly outperformed random guessing (*p*-values < 1 × 10^−6^) in distinguishing responders from non-responders. However, no significant differences were found between these classifiers in their ability to differentiate between the two responder groups (*p*-values > 0.190).

## 4. Discussion

Radiomics is a rapidly evolving field in medical imaging that involves the extraction and analysis of a large number of quantitative features from oncological images, such as CT scans, MRI images, or PET scans, among other radiographic data [42,43,44,45]. These features go beyond what the human eye can perceive and comprise characteristics corresponding, typically, to shape, size, texture, and intensity patterns within the image region of interest [46]. Radiomics operates on the principle that the extraction of quantitative imaging characterization can provide a comprehensive view of intra-tumoral phenotypic and genetic heterogeneity and thus help better understand the molecular and functional basis of the underlying disease [14,47]. Radiomics has found applications in many different medical realms, but it is particularly prominent in clinical oncology, where it holds the potential to facilitate personalized treatment approaches by tailoring therapies based on the specific radiomics profile of a patient’s disease [46]. The current study aimed to assess the capacity of longitudinal FDG-PET radiomics to distinguish between LACC patients who responded to treatment and those who did not. The clinical importance of being able to predict response to treatment early in LACC has significant clinical implications, spanning treatment customization, toxicity reduction, survival improvement, and long-term patient prognosis.

When comparing the best-selected features, those derived from the interim scans were able to differentiate the two responder groups at statistically significant levels. In contrast, the best-selected features from the pretreatment scans showed no significant differences between the groups. This suggests a notable difference in the predictive power of radiomics features from interim scans compared to those from pretreatment scans in distinguishing between responders and non-responders. Interim scans, taken midway through treatment, may capture early metabolic changes or responses to chemoradiation that are absent in pretreatment PET scans, providing more treatment-relevant radiographic information for distinguishing between the groups. In contrast, the lack of significant differences in pretreatment features suggests that baseline radiographic characteristics are less predictive of treatment response than those observed during treatment. The differences in radiomics features between pretreatment and interim scans open up new avenues for research into why certain features become significant only during treatment and how they can be leveraged to improve early response prediction. Further exploration of these aspects could provide deeper insights into the role of imaging features at different treatment stages and their implications for patient management and outcomes in LACC.

The comparison between the two predictive models—one developed using the top two radiomics features from pretreatment scans and the other from interim scans—further reveals a considerable difference in their ability to distinguish between responder groups. Specifically, the AUC of the mean ROC curve for the model based on pretreatment scans is 0.8529, while the AUC of the mean ROC curve for the model using interim scans reaches 0.9420. This notable difference underscores the superior performance of the model based on interim scans, demonstrating that mid-treatment imaging offers greater predictive power for assessing treatment response compared to baseline scans. Although the difference in AUC values between the two models is substantial, it was not found to be statistically significant. This is primarily due to the inadequate number of patients in the current study cohort, which limited the study’s statistical power, even though clinically relevant differences are conspicuous. A post hoc power analysis indicated that a sample size of at least 250 patients per group would be needed to achieve 80% power to detect a significant difference at the 5% alpha level [48].

The comparison between radiomics parameters extracted from pretreatment and interim scans, whether evaluated individually for differentiating responder groups or collectively in predictive models, shows improved predictive value associated with interim scans. This enhanced predictive value from interim scans could have important implications for managing patients with LACC, a challenging condition due to its heterogeneous nature and variable response to standard chemoradiation therapy [49]. First, it allows for more personalized treatment. Patients identified early as responders may benefit from de-escalated treatment regimens, reducing exposure to toxic therapies without compromising efficacy. Conversely, those classified as potential non-responders may be candidates for intensified treatments or alternative therapies. By avoiding unnecessary treatments in patients who are unlikely to respond, risks of treatment-related toxicity could be reduced. This is particularly crucial in LACC, where chemoradiation can cause significant side effects [50]. Furthermore, improved predictive values may aid in reliably identifying LACC patients with favorable outcomes. This can help optimize the allocation of valuable medical resources, ensuring that treatment is directed toward patients most likely to benefit. In addition, with better predictive value, follow-up protocols can be tailored based on the predicted response, such as patients identified as non-responders requiring more frequent monitoring for potential disease progression.

While the results are promising, several limitations should be considered when interpreting the findings. The primary limitation is the relatively small number of patients included in the study, as previously mentioned. A small sample size can limit the statistical power of the findings and increase the risk of overfitting [51]. To mitigate this issue, n-fold cross-validation was employed, which creates multiple train–test splits to provide a more comprehensive evaluation of model performance across different data subsets. Additionally, model performance was assessed by averaging the ROC curves from each cross-validation trial, which helps reduce the variability that might arise from a single train–test split and better estimates how well the model is likely to perform on new, unseen data [39]. Although these measures help lessen the impact of a small sample size, they do not entirely eliminate it. Therefore, the findings should be interpreted with caution, and further validation with more extensive, independent datasets is necessary. Moreover, the analysis was conducted retrospectively on a single patient cohort from one institution. Consequently, confounders inherent to the observational study design were not addressed [52]. Thus, prospective validation of the results is needed, ideally in a multicenter clinical trial setting.

## 5. Conclusions

The enhanced predictive capability of longitudinal radiomics in differentiating between LACC patients who responded to chemoradiation and those who did not underscores the potential of interim PET for predictive risk stratification of treatment response. Longitudinal radiomics could be crucial in personalized medicine and improving therapeutic outcomes for LACC.

## Figures and Tables

**Table 1:** Radiomics categories and features analyzed in the study.

Category	Feature
Gray-level Co-occurrence Matrix (GLCM)	Autocorrelation, Cluster prominence, Cluster shade, Cluster tendency, Contrast, Correlation, Difference average, Difference entropy, Inverse difference, Difference variance, Inverse difference moment, Inverse difference moment normalized, Inverse difference normalized, Inverse variance, Joint entropy, Informational measure of correlation 1, Informational measure of correlation 2, Joint average, Joint energy, Maximal correlation coefficient, Maximum probability, Sum average, Sum entropy, Sum squares
Gray-level Dependence Matrix (GLDM)	Dependence entropy, Dependence nonuniformity, Dependence nonuniformity normalized, Dependence variance, Gray-level nonuniformity, Gray-level variance, High gray-level emphasis, Large dependence emphasis, Large dependence high gray-level emphasis, Large dependence low gray-level emphasis, Low gray-level emphasis, Small dependence emphasis, Small dependence high gray-level emphasis, Small dependence low gray-level emphasis
Gray-level Run Length Matrix (GLRLM)	Gray-level nonuniformity, Gray-level nonuniformity normalized, Gray-level variance, High gray-level run emphasis, Long run emphasis, Long run high gray-level emphasis, Long run low gray-level emphasis, Low gray-level run emphasis, Run entropy, Run length nonuniformity, Run length nonuniformity normalized, Run percentage, Run variance, Short run emphasis, Short run high gray-level emphasis, Short run low gray-level emphasis
Neighboring Gray Tone Difference Matrix (NGTDM)	Busyness, Coarseness, Complexity, Contrast, Strength

## Data Availability

All data are provided in the paper.

## References

[1] Zhang X., Zeng Q., Cai W., Ruan W. (2021). Trends of cervical cancer at global, regional, and national level: Data from the Global Burden of Disease study 2019. BMC Public Health.

[2] Barwick T.D., Taylor A., Rockall A. (2013). Functional imaging to predict tumor response in locally advanced cervical cancer. Curr. Oncol. Rep..

[3] Yang F., Thomas M.A., Dehdashti F., Grigsby P.W. (2013). Temporal analysis of intratumoral metabolic heterogeneity characterized by textural features in cervical cancer. Eur. J. Nucl. Med. Mol. Imaging.

[4] Rohren E.M., Turkington T.G., Coleman R.E. (2004). Clinical applications of PET in oncology. Radiology.

[5] Herrera F.G., Prior J.O. (2013). The role of PET/CT in cervical cancer. Front. Oncol..

[6] Roberts C., Liyanage S.H., Harry V.N., Rockall A.G. (2011). Functional imaging for assessing tumor response in cancer of the cervix. Womens Health.

[7] Yang F., Grigsby P.W. (2012). A segmentation framework towards automatic generation of boost subvolumes for FDG-PET tumors: A digital phantom study. Eur. J. Radiol..

[8] Davidson S.E., West C.M.L., Roberts S.A., Hendry J.H., Hunter R.D. (1990). Radiosensitivity testing of primary cervical-carcinoma—Evaluation of intra-tumor and inter-tumor heterogeneity. Radiother. Oncol..

[9] Gillies R.J., Kinahan P.E., Hricak H. (2016). Radiomics: Images Are More than Pictures, They Are Data. Radiology.

[10] Tomaszewski M.R., Gillies R.J. (2021). The biological meaning of radiomic features. Radiology.

[11] Apostolova I., Ego K., Steffen I.G., Buchert R., Wertzel H., Achenbach H.J., Riedel S., Schreiber J., Schultz M., Furth C. (2016). The asphericity of the metabolic tumour volume in NSCLC: Correlation with histopathology and molecular markers. Eur. J. Nucl. Med. Mol. Imaging.

[12] Bundschuh R.A., Dinges J., Neumann L., Seyfried M., Zsótér N., Papp L., Rosenberg R., Becker K., Astner S.T., Henninger M. (2014). Textural parameters of tumor heterogeneity in 18F-FDG PET/CT for therapy response assessment and prognosis in patients with locally advanced rectal cancer. J. Nucl. Med..

[13] Grabinska K., Pelak M., Wydmanski J., Tukiendorf A., d’Amico A. (2015). Prognostic value and clinical correlations of 18-fluorodeoxyglucose metabolism quantifiers in gastric cancer. World J. Gastroenterol..

[14] Yang F., Young L.A., Johnson P.B. (2018). Quantitative radiomics: Validating image textural features for oncological PET in lung cancer. Radiother. Oncol..

[15] Alderuccio J.P., Kuker R.A., Yang F., Moskowitz C.H. (2023). Quantitative PET-based biomarkers in lymphoma: Getting ready for primetime. Nat. Rev. Clin. Oncol..

[16] Burchardt E., Bos-Liedke A., Serkowska K., Cegla P., Piotrowski A., Malicki J. (2023). Value of [18F] FDG PET/CT radiomic parameters in the context of response to chemotherapy in advanced cervical cancer. Sci. Rep..

[17] Lucia F., Visvikis D., Desseroit M.-C., Miranda O., Malhaire J.-P., Robin P., Pradier O., Hatt M., Schick U. (2018). Prediction of outcome using pretreatment 18 F-FDG PET/CT and MRI radiomics in locally advanced cervical cancer treated with chemoradiotherapy. Eur. J. Nucl. Med. Mol. Imaging.

[18] Ferreira M., Lovinfosse P., Hermesse J., Decuypere M., Rousseau C., Lucia F., Schick U., Reinhold C., Robin P., Hatt M. (2021). [18 F] FDG PET radiomics to predict disease-free survival in cervical cancer: A multi-scanner/center study with external validation. Eur. J. Nucl. Med. Mol. Imaging.

[19] Mayr N., Yuh W., Bowen S., Harkenrider M., Knopp M., Lee E., Leung E., Lo S., Small W., Wolfson H. (2023). Cervical Cancer—Tumor Heterogeneity: Serial Functional and Molecular Imaging Across the Radiation Therapy Course in Advanced Cervical Cancer (Version 1). Cancer Imaging Arch..

[20] Clark K., Vendt B., Smith K., Freymann J., Kirby J., Koppel P., Moore S., Phillips S., Maffitt D., Pringle M. (2013). The Cancer Imaging Archive (TCIA): Maintaining and operating a public information repository. J. Digit. Imaging.

[21] Bowen S.R., Yuh W.T.C., Hippe D.S., Wu W., Partridge S.C., Elias S., Jia G., Huang Z., Sandison G.A., Nelson D. (2018). Tumor radiomic heterogeneity: Multiparametric functional imaging to characterize variability and predict response following cervical cancer radiation therapy. J. Magn. Reson. Imaging.

[22] Schaue D., Micewicz E.D., Ratikan J.A., Xie M.W., Cheng G., McBride W.H. (2015). Radiation and inflammation. Semin. Radiat. Oncol..

[23] Weichselbaum R.R., Liang H., Deng L., Fu Y.-X. (2017). Radiotherapy and immunotherapy: A beneficial liaison?. Nat. Rev. Clin. Oncol..

[24] Vadi S.K., Mittal B.R. (2021). FDG PET/CT in Treatment Response Evaluation of Gynecological Malignancies. Atlas of Clinical PET-CT in Treatment Response Evaluation in Oncology.

[25] Eminowicz G., Hall-Craggs M., Diez P., McCormack M. (2016). Improving target volume delineation in intact cervical carcinoma: Literature review and step-by-step pictorial atlas to aid contouring. Pract. Radiat. Oncol..

[26] Leijenaar R.T.H., Nalbantov G., Carvalho S., van Elmpt W.J.C., Troost E.G.C., Boellaard R., Aerts H.J.W.L., Gillies R.J., Lambin P. (2015). The effect of SUV discretization in quantitative FDG-PET Radiomics: The need for standardized methodology in tumor texture analysis. Sci. Rep..

[27] Johnson P.B., Young L.A., Lamichhane N., Patel V., Chinea F.M., Yang F. (2017). Quantitative imaging: Correlating image features with the segmentation accuracy of PET based tumor contours in the lung. Radiother. Oncol..

[28] Haralick R.M. (1979). Statistical and structural approaches to texture. Proc. IEEE.

[29] Yang F., Dogan N., Stoyanova R., Ford J. (2018). Evaluation of radiomic texture feature error due to MRI acquisition and reconstruction: A simulation study utilizing ground truth. Phys. Med..

[30] Galloway M.M. (1975). Texture analysis using gray level run lengths. Comput. Graphic. Image Process..

[31] Ford J., Dogan N., Young L., Yang F. (2018). Quantitative Radiomics: Impact of Pulse Sequence Parameter Selection on MRI-Based Textural Features of the Brain. Contrast Media Mol. Imaging.

[32] Stoecker W.V., Chiang C.S., Moss R.H. (1992). Texture in skin images: Comparison of three methods to determine smoothness. Comput. Med. Imaging Graph..

[33] Sun C., Wee W.G. (1983). Neighboring gray level dependence matrix for texture classification. Comput. Vis. Graph. Image Process..

[34] Altmann A., Toloşi L., Sander O., Lengauer T. (2010). Permutation importance: A corrected feature importance measure. Bioinformatics.

[35] Breiman L. (2001). Random forests. Mach. Learn..

[36] Dau H.A., Bagnall A., Kamgar K., Yeh C.-C.M., Zhu Y., Gharghabi S., Ratanamahatana C.A., Keogh E. (2019). The UCR time series archive. IEEE/CAA J. Autom. Sin..

[37] Kohavi R. A study of cross-validation and bootstrap for accuracy estimation and model selection. Proceedings of the 14th International Joint Conference on Artificial Intelligence.

[38] Kruskal W.H., Wallis W.A. (1952). Use of ranks in one-criterion variance analysis. J. Am. Stat. Assoc..

[39] Fawcett T. (2006). An introduction to ROC analysis. Pattern Recognit. Lett..

[40] Hosmer D.W., Lemeshow S., Sturdivant R.X. (2013). Applied Logistic Regression.

[41] DeLong E.R., DeLong D.M., Clarke-Pearson D.L. (1988). Comparing the areas under two or more correlated receiver operating characteristic curves: A nonparametric approach. Biometrics.

[42] Aerts H.J., Velazquez E.R., Leijenaar R.T., Parmar C., Grossmann P., Carvalho S., Cavalho S., Bussink J., Monshouwer R., Haibe-Kains B. (2014). Decoding tumour phenotype by noninvasive imaging using a quantitative radiomics approach. Nat. Commun..

[43] Lambin P., Rios-Velazquez E., Leijenaar R., Carvalho S., van Stiphout R.G., Granton P., Zegers C.M., Gillies R., Boellard R., Dekker A. (2012). Radiomics: Extracting more information from medical images using advanced feature analysis. Eur. J. Cancer.

[44] Yang F., Ford J.C., Dogan N., Padgett K.R., Breto A.L., Abramowitz M.C., Dal Pra A., Pollack A., Stoyanova R. (2018). Magnetic resonance imaging (MRI)-based radiomics for prostate cancer radiotherapy. Transl. Androl. Urol..

[45] van Griethuysen J.J.M., Fedorov A., Parmar C., Hosny A., Aucoin N., Narayan V., Beets-Tan R.G.H., Fillion-Robin J.-C., Pieper S., Aerts H.J.W.L. (2017). Computational Radiomics System to Decode the Radiographic Phenotype. Cancer Res..

[46] van Timmeren J.E., Cester D., Tanadini-Lang S., Alkadhi H., Baessler B. (2020). Radiomics in medical imaging-”how-to” guide and critical reflection. Insights Imaging.

[47] Park J.E., Kim H.S. (2018). Radiomics as a Quantitative Imaging Biomarker: Practical Considerations and the Current Standpoint in Neuro-oncologic Studies. Nucl. Med. Mol. Imaging.

[48] López-Ratón M., Rodríguez-Álvarez M.X., Cadarso-Suárez C., Gude-Sampedro F. (2014). Optimal Cutpoints: An R package for selecting optimal cutpoints in diagnostic tests. J. Stat. Softw..

[49] Rose P.G., Bundy B.N. (2002). Chemoradiation for locally advanced cervical cancer: Does it help?. J. Clin. Oncol..

[50] Keys H.M., Bundy B.N., Stehman F.B., Muderspach L.I., Chafe W.E., Suggs C.L., Walker J.L., Gersell D. (1999). Cisplatin, radiation, and adjuvant hysterectomy compared with radiation and adjuvant hysterectomy for bulky stage IB cervical carcinoma. N. Engl. J. Med..

[51] Vittinghoff E., McCulloch C.E. (2007). Relaxing the rule of ten events per variable in logistic and Cox regression. Am. J. Epidemiol..

[52] Concato J., Shah N., Horwitz R.I. (2017). Randomized, controlled trials, observational studies, and the hierarchy of research designs. Research Ethics.

